# The Use of Lisdexamfetamine to Treat ADHD in a Patient with Stimulant (Methamphetamine) Use Disorder

**DOI:** 10.1155/2023/5574677

**Published:** 2023-08-14

**Authors:** J. Levine, H. Swanson

**Affiliations:** ^1^University of Minnesota Medical School, Minneapolis, MN, USA; ^2^VA Medical Center, Minneapolis, MN, USA

## Abstract

**Introduction:**

The treatment of attention deficit hyperactivity disorder (ADHD) with stimulants among patients with stimulant use disorder carries concern for efficacy and exacerbation of addictive behaviors. Lisdexamfetamine is a unique stimulant used to treat ADHD with a lower abuse potential compared to other stimulants, as the medication is the only prodrug in its class. To our knowledge, there are no reports in the literature of the use of lisdexamfetamine to treat ADHD in patients with stimulant use disorder.

**Methods:**

We present a 33-year-old male with a history of stimulant (methamphetamine) use disorder, who was found to have long-standing ADHD. The patient was treated with lisdexamfetamine 30 mg, which was increased and sustained at 40 mg.

**Results:**

The patient reported significant improvement in focus, concentration, calmness, organization of thoughts, and less of a tendency to procrastinate. Additionally, he denied exacerbation of anxiety or sleep disturbances. He reported his cravings for stimulants were significantly decreased. After 2 months of treatment, he had moved out from his sober living facility, started a new job, and gained a promotion. He had no use of illicit substances, which was proven by routine urine drug screens.

**Conclusion:**

Our patient's ADHD was successfully treated with lisdexamfetamine. Not only did the patient's ADHD symptoms improve, but his cravings for stimulants were relieved. ADHD is common among patients with stimulant use disorder. Patients with ADHD and stimulant use disorder should not necessarily forgo pharmacologic treatment with stimulants for concerns of abuse. Due to its unique pharmacokinetic profile, lisdexamfetamine is a feasible treatment for patients with ADHD and a history of stimulant use disorder.

## 1. Introduction

Attention deficit hyperactivity disorder (ADHD) is a prevalent comorbidity among patients who have problematic use of methamphetamine and other stimulants [1]. Despite this correlation, literature evaluating pharmacologic intervention of ADHD in adults with stimulant use disorder is limited [2]. The Adult ADHD Self-Report Scale (ASRS) is a validated screening tool that allows clinicians to identify adults with ADHD by evaluation of executive functioning deficits that are not explicitly stated in the Diagnostic and Statistical Manual of Mental Disorders, Fifth Edition (DSM-5) criteria [3]. The treatment of ADHD with stimulants in patients with a history of stimulant abuse carries concerns regarding both the efficacy of treatment, as well as concerns of misuse and/or precipitation of addictive behaviors [4]. Literature review yields both (1) studies evaluating treatment of coexisting ADHD and stimulant use disorder [2], as well as (2) the use of prescription stimulants in the treatment of stimulant use disorder [4, 5]. However, to our knowledge, no studies have evaluated treatment of ADHD in adults with stimulant use disorder with lisdexamfetamine.

Amphetamine-type stimulants, including methamphetamine, are the fastest rising drug of abuse worldwide [6]. They were initially produced over a century ago and were used for a variety of medical applications, as well as in the military applications during the Second World War [7]. Today, pharmaceutical methamphetamine is still produced, though its use in clinical medicine has largely been supplanted by newer stimulant formulations [8].

Amphetamines, specifically mixed amphetamine/dextroamphetamine salts, are perhaps the most widely prescribed medication for adults with ADHD. A main concern of treating ADHD with fast-acting/immediate-release formulations in patients with comorbid stimulant use disorder is the potential for misuse, diversion, and exacerbation of addictive behaviors. Studies have shown that fast-acting stimulants may pose a greater risk for abuse potential compared to longer-acting agents [9]. Thus, a longer-acting amphetamine may be an ideal medication option in the treatment of adults with concurrent ADHD and stimulant use disorder. This case presentation focuses on the treatment of an adult with comorbid ADHD and stimulant use disorder using lisdexamfetamine.

## 2. Case Presentation

A 33-year-old male presented to an outpatient psychiatric clinic for evaluation. He endorsed a history of undiagnosed anxiety, methamphetamine use, and multiple legal issues, including various forms of theft. He admitted being externally motivated to seek mental health/substance use disorder treatment because of his involvement with the justice system. However, in addition, he noted the presence of internal motivation—something that is quite new for him. He stated: “I want to do this for myself. I'm tired of living like this.” At the time of our first clinic visit, he reported that he had not used methamphetamine for the past 2 months. Prior to then, he reported using methamphetamine “whenever he could get his hands on it,” ranging from daily to weekly use. At the time of initial evaluation, he did not endorse regular cravings for stimulants. He was unemployed and resided in a sober living facility.

The patient reported having issues maintaining attention and concentration since his childhood. ADHD had been diagnosed, but no formal testing results were available. Previous treatment modalities include psychotherapy, but he was never prescribed medications to address his attention and concentration issues. Around this time, he began engaging in illegal behaviors, such as stealing vehicles (four-wheelers, snowmobiles, cars) in his adolescence—all the way up to semitrailers and heavy machinery (e.g., bulldozer) later in his teenage years. He endorsed a tendency to procrastinate, frequently forgetting things, and often spending excessive amounts of time double checking various things (e.g., emails, that things in his household (e.g., oven, shower) are turned off, and ensuring he has his all of his personal belongings) before leaving his residence. He also reported difficulty sitting still and interrupting others when they are busy or when he loses interest or becomes bored.

His stimulant use history began in high school. In addition, he has had periods of heavy alcohol use, which is now in sustained remission after receiving his second driving under the influence ticket and deciding to quit drinking. He regularly uses tobacco (averages one pack of cigarettes per day) and marijuana. No other substances abuse was reported.

Although he has received various negative consequences, he denies feeling that his methamphetamine use was problematic. In fact, he cites various benefits from his use. For example, methamphetamine allows him to “feel calm,” helps “quiet racing thoughts,” and enables him to focus on specific tasks instead of “bouncing around.” Essentially, he views his methamphetamine use as a form of “self-medication” for his mental health symptoms—namely his struggles with attention and concentration but also subsyndromal anxiety and mood.

During his initial evaluation, the patient was asked to fill out the ASRS-V1.1 adult ADHD self-reported scale. For part A, 6/6 of the darkly shaded boxes were marked and if 4+ darkly shaded boxes are marked, the results are highly consistent with ADHD. Additionally, 7/12 of the darkly shaded boxes were marked in part B, which is not as diagnostic as part A but gives additional cues and probes into the patient's symptoms. The results of the assessment were highly consistent with ADHD.

At the end of the initial psychiatric evaluation, the patient was started on lisdexamfetamine 30 mg daily and instructed to follow-up within 1 month. Even before this next scheduled visit, he reported numerous positive benefits from the medication, including improved focus, concentration, clarity of speech, improvement in organization of thought process, less tendency to procrastinate, etc. He denied exacerbation of underlying anxiety (rather, he noted improved sense of calm overall) nor did he have sleep disturbances. His cravings for “illicit” stimulants were significantly decreased. Unfortunately, he was not able to continue lisdexamfetamine, as his sober living facility later informed him that controlled substances (such as stimulants, Drug Enforcement Administration (DEA) schedule II) were not allowed to be continued while in residence. Therefore, this medication was stopped after approximately 1 week. At this time, we opted to try a nonstimulant, wakefulness-promoting medication, armodafinil, for the remainder of his time at the facility.

At his initial follow-up visit, he informed clinic staff that he had since moved out of the sober living facility and was interested in restarting lisdexamfetamine. Upon discharge, he had resumed lisdexamfetamine, utilizing the remainder of the medication left over from the initial prescription. He shared that he opted not to start the armodafinil, citing concerns after researching the medication and reading the list of side effects. He said he often has poor reactions to medications. He gained employment as an assistant manager of a retail store and had been considering starting his own business. He reported the same benefits of lisdexamfetamine as described above, but started to notice that the medication was wearing off sooner than was ideal—therefore, the dose was titrated to 40 mg daily with the hopes of sustaining the clinical benefit longer in the day.

At a follow-up appointment 2 months later, the patient reported that the increased dose of lisdexamfetamine had been working well and was pleased to share that he hadn't missed any days of work. He has enjoyed various positive changes, including a promotion at work from assistant manager to general manager. Eight months after starting treatment with lisdexamfetamine, he continued to endorse abstinence from “illicit” stimulants, which is corroborated by intermittent urine toxicology reports every 6 weeks, as well as the positive effects noted above.

## 3. Discussion

In adult patients, the DSM-5 criteria for the diagnosis of ADHD require at least five symptoms relating to inattention, hyperactivity, and impulsivity for at least 6 months [10]. The patient described having a history of forgetfulness, issues completing tasks, difficulty in sustaining mental efforts, being easily distracted, and hyperactivity and impulsivity in over two settings since prior to the age of 12 years. His symptoms met the DSM-5 criteria for ADHD.

According to the 2019 World Drug Report, there are about 25 million stimulant abusers worldwide and this number is increasing [11]. In a cross-sectional study of 134 patients with a history of methamphetamine, 10.4% were found to have adult ADHD [12]. Since there are a large number of patients with stimulant use disorder worldwide and many of them may have concomitant ADHD, this study could represent a large patient population.

Careful consideration for the pharmacologic treatment of this patient's ADHD was required, as misuse of prescribed stimulants is often of concern in patients who have exhibited problematic substance use in the past. Atomoxetine, a presynaptic norepinephrine transport inhibitor, is often considered in patients where stimulant diversion is a problem [13]. Since it is not classified as a stimulant, concern for abuse/misuse tends to be quite low. However, atomoxetine is not without risks: it (like many other psychotropic medications) carries a “black box” warning for increased suicidal ideation, among other potential issues [14]. In addition, atomoxetine has been found to be less effective for symptom management in adults with ADHD (as compared to the other available stimulant medications) [5]. Literature comparing the use of lisdexamfetamine and atomoxetine in adults with ADHD is sparse. A study using US commercial claims databases compared 3,392 patients who discontinued methylphenidate and started either lisdexamfetamine or atomoxetine as second-line treatments. The study found that patients treated with lisdexamfetamine had higher treatment adherence and lower discontinuation rates as compared to those treated with atomoxetine [15].

Lisdexamfetamine is a unique stimulant option, as it is the only available stimulant that is classified as a “prodrug” [4]. Lisdexamfetamine is primarily absorbed in the small intestines. As a prodrug, the medication is inactive until it is metabolized into its active form, dextroamphetamine, through enzymatic hydrolysis, which occurs in the blood after entering the portal circulation [16]. Studies have shown that due to its pharmacokinetic profile, lisdexamfetamine has a lower abuse-related drug-liking score compared to immediate-release dextroamphetamine [17]. In addition, various studies have demonstrated the efficacy of lisdexamfetamine in the treatment of adults with ADHD [14, 15, 18]. Additionally, lisdexamfetamine may be effective in the treatment of stimulant (methamphetamine) use disorder [4, 19, 20]. Mixed literature exists regarding the validity of stimulant treatment of ADHD exacerbating cravings and addictive behaviors in patients with stimulant use disorder [21]. Due to its low abuse potential and high efficacy, lisdexamfetamine is an ideal treatment option in this patient population.

In this particular clinical case, lisdexamfetamine was found to be an effective medication for the treatment of this patient's ADHD and did not exacerbate his underlying stimulant (methamphetamine) use disorder. Rather, the medication was found to be beneficial for substance use disorder: he reported significant reduction in cravings for methamphetamine or other stimulants and has not returned to “illicit” stimulant use since establishing care with our clinic.

The main limitation to this study is that it is limited to one patient. Thus, the implications of effective treatment of ADHD in patients with stimulant use disorder with lisdexamfetamine may not be applicable to the population. Future studies, such as a randomized controlled trial, may be warranted as a large number of patients worldwide may benefit from this novel treatment. Additionally, a longer follow-up time could be utilized to demonstrate a longer lasting therapeutic benefit.

## 4. Conclusion

Given the aforementioned factors, lisdexamfetamine is uniquely poised to treat ADHD in the context of patients with a history of stimulant use disorders. Lisdexamfetamine is an effective and well-tolerated medication for the treatment of ADHD with a lower abuse potential than faster-acting stimulants. Therefore, it is our opinion that lisdexamfetamine should be strongly considered when treating a patient with comorbid ADHD and stimulant use disorder. Further research is warranted to better understand the efficacy of lisdexamfetamine in this patient population.

## Data Availability

The data that support the findings of this article are available from the corresponding author on reasonable request.
